# Inferring transposons activity chronology by TRANScendence – TEs database and *de-novo* mining tool

**DOI:** 10.1186/s12859-017-1824-4

**Published:** 2017-10-16

**Authors:** Michał Piotr Startek, Jakub Nogły, Agnieszka Gromadka, Dariusz Grzebelus, Anna Gambin

**Affiliations:** 1 Institute of Informatics, University of Warsaw, Banacha 2, Warsaw, 02097 Poland; 2 Institute of Plant Biology and Biotechnology, University of Agriculture in Kraków, 29 Listopada 54, Kraków, 31425 Poland

**Keywords:** Transposable elements, Evolutionary history, Hill-climbing algorithm

## Abstract

**Background:**

The constant progress in sequencing technology leads to ever increasing amounts of genomic data. In the light of current evidence transposable elements (TEs for short) are becoming useful tools for learning about the evolution of host genome. Therefore the software for genome-wide detection and analysis of TEs is of great interest.

**Results:**

Here we describe the computational tool for mining, classifying and storing TEs from newly sequenced genomes. This is an online, web-based, user-friendly service, enabling users to upload their own genomic data, and perform *de-novo* searches for TEs. The detected TEs are automatically analyzed, compared to reference databases, annotated, clustered into families, and stored in TEs repository. Also, the genome-wide nesting structure of found elements are detected and analyzed by new method for inferring evolutionary history of TEs.

We illustrate the functionality of our tool by performing a full-scale analyses of TE landscape in *Medicago truncatula* genome.

**Conclusions:**

TRANScendence is an effective tool for the de-novo annotation and classification of transposable elements in newly-acquired genomes. Its streamlined interface makes it well-suited for evolutionary studies.

## Background

Transposable elements (TEs) are genetic entities capable of changing their genomic localization. They constitute a significant portion of eucaryotic genomes. Two classes of transposable elements are commonly recognized. Class I gropus retrotransposons, i.e. elements transposing via an RNA intermediate using a ‘copy and paste’ mechanism. Class II comprises DNA transposons that are being physically excised from the donor site upon mobilization and transpose through a ‘cut and paste’ mechanism.

The role of transposable elements in genome evolution was previously marginalized and underestimated. Nowadays these ubiquitous and widespread mobile genetic entities are no longer perceived as just ‘junk’ DNA. There is a molecular evidence that mobile elements can affect the evolution of their host genomes. They might be even consider as driving force behind species evolution and speciation [1, 2]. Their fractions in different taxons, like plants, are very variable, as low as 3% in small genomes and as high as 85% in larger genomes. It might even indicate that the genome size is a linear function of transposable element content.

The advent of sequencing techniques brings affluence of genomic data. Unfortunately, most of them was not investigated for transposable elements. The main obstacle here is the lack of freely available and easily to use software for TEs detection and annotation. This clearly inhibits the scientific progress in the field of comparative genomics of transposable elements. The studies of TEs consist of several tasks, like searches, classification, annotation, and so on. Depending of type of elements there are specific methods which are sensitive for them [3], e.g. structure-based methods [4] for long terminal repeat (LTR) retrotransposons [5]. A variety of tools performing each from above mentioned steps have been proposed. The comprehensive list of available computational resources prepared by Bergman Lab (http://bergmanlab.ls.manchester.ac.uk/) contains more then 120 elements.

Aiming in the complete analysis of TE landscape one have to design the appropriate pipeline consisted of several tools. One such solution, proposed for de-novo TEs analysis is called REPET [6]. It combines variety of approaches to search for new repeats and annotate them, mostly by comparing found repeats to consensus sequences stored in the Repbase [7]. Unfortunately, to use the pipeline one has to install and configure each component separately.

### Our results

Our first goal was to integrate REPET pipeline with smart and malleable TEs repository and to provide the user friendly web-interface enabling complete de-novo TE analysis.

Proposed tool, called TRANScendence in contrast with previous methods, is totally automatic. However, the results can be also curated manually if desired. TRANScendence is not only transposon mining tool. More importantly, it is able to perform different quantitative and qualitative analysis. The TEs are classified into their orders, superfamilies and families which allows to track the evolution of each TE family in the host genomes. Moreover, we focused on reconstruction of the chronology of TEs families activity. Proposed algorithms examine the regions abundant of transposons nested one in another. Then the nesting structure suggest the evolutionary history of TE families activity.

### Organization of the paper

“Methods” section starts with the description of TE interruption graph, based on which the chronology of TE families activity is inferred. Then the complexity of the problem is analyzed and efficient algorithms for reconstruction of TE evolutionary history are presented. Last subsection presents the functionality of our web service for mining and analysing TEs and justify the its usefulness by analyzing the TE landscape of *Medicago truncatula* genome. “Results and discussion” section contains validation of proposed algorithms on simulated and real datasets (*Drosophila melanogaster* and human genome). Concluding section discusses the possible directions for further research.

### Availability

Software has been already used in several scientific projects [8, 9]. To support future evolutionary studies, we present our tool as free and flexible web service available at: http://bioputer.mimuw.edu.pl/transcendence.

## Methods

High density of TEs, especially in plant genomes, is the result of the periodic invasions and bursts of activity of different TEs over millions of years. The insertion activity may cause the splitting of TEs already existing in the genome into noncontiguous fragments separated by the sequence of newly inserted elements. The identification of nested transposable elements is important for evolutionary comparisons among various regions of the genome. For human genome the chronology of TE families based on the TEs nesting was studied in [1]. For highly repetitive plants genomes the TEnest tool have been proposed [10] for visual representation of TE integration history.

The latter tool focuses on long terminal repeat (LTR) retrotransposons and more importantly is no longer unavailable to use at PlantGDB site. In our service we implemented the nesting repeats identification scheme analogous to the approach previously applied to human genome [1]. All found TEs nesting are displayed in a graphical format, and may be reviewed online. In addition to that, a graph of nesting dependencies between TE families is constructed, along with interruption matrix.

By TE interruption matrix we mean a matrix such that rows and columns represent TE families and the values count the interruption events between two families.

TE dispersal in the genomes can be characterized by a period of transpositional activity during which TE copies are spread throughout the genome, followed by gradual inactivation by loss of transpositional ability due to silencing mechanimsms protecting integrity of the host genome. The remnant TE copies remain behind and could be degraded over time by various mutation events, including insertions of newer TEs. The result is that the older TE will become interrupted by newer elements but will not be inserted into newer elements. On the other hand, the newer elements, with a relatively recent period of activity, will be inserted into older ones, but will not be interrupted by older elements. Elements of intermediate age will be both inserted into older elements and fragmented by newer elements.

If we consider the interruption matrix where TE families are in chronological order it should be close to lower triangular matrix. For a general matrix the task of finding such an order that minimize the number of insertions in the upper triangle is *NP*-complete problem. Therefore we propose a heuristic search method to infer the chronology of TEs activity. Then we demonstrate how the method works for the interruption graphs generated using specific model of TE evolution. The outcome of our approach on real data (human genome and *Drosophila melanogaster* genome) is also presented and compared to very limited studies on TEs chronology.

### Inferring TEs evolutionary history from the interruption graph

We can treat an interruption matrix as adjacency matrix of some directed graph *G*. Each vertex corresponds to some TE family and vice versa. In this interpretation the edge from vertex *v* to vertex *w* describes an event of interruption of TE belonging to family *w* by TE from family *v*. As we allow multi edges between a given pair of vertices, *G* is a multigraph. The more edges between two vertices, the greater is the number of times the interruption event took place between them. The general idea is that edges point from younger TE families to older ones, thus, establishing a partial ordering. This simple image is complicated by the fact that two TE families could have overlapping periods of activity (and so, they could both insert into each other) and also by noise caused by genomic rearrangements, as well as that introduced during computational detection and annotation of TEs.

Summarizing, interruption matrix *M* is a nonnegative integer matrix which rows and columns representing TE families. Namely, *M*[ *v*][*w*]=*c*, if and only if the event of insertion TE from family *v* into some TE belonging to family *w* took place *c* times.

We denote the graph induced by interruption matrix *M* by *G*_*M*_. TE families could be numerated from 0 to *n*, so we can talk about permutation *σ* of the families. We say that family *v* is before family *w*, if and only if *σ*(*v*)<*σ*(*w*).

Hence, *σ* defines the chronology of families. In the sequel, we try to find such an order of families that minimize the number of back edges in *G*_*M*_, so we would like to minimize function *f*, given by:

$$f(\sigma) = \left|\{(v, w) \in G_{M}\ \mid \sigma(v) > \sigma(w)\}\right| $$

It is easy to prove that the following decision problem is NP-complete (reduction from FEEDBACKARCSET [11]): *given interruption matrix M and positive integer k. Decide whether there exists a permutation σ such that f(x03C3;)≤k.*

Main idea of the simplest approach, called quasi-topological sort is the following: the lower in-degree of a given vertex the fewer of times the corresponding TE family was interrupted, so as a consequence the newer the TE family is. Thus, we can apply topological approach to sort the vertices. We iteratively take vertex with the lowest in-degree, represent the newest the TE and remove it from the graph.

We can slightly improve quasi-topological approach by performing decomposition of *G* into strongly connected components. It could be done using *Tarjan* algorithm [12] in linear time. Having computed the components, we can order them. After that, we create a graph of strongly connected components, which is a DAG (directed acyclic graph), denote it by *H*. Then, it is enough to sort each strongly connected component and then to concatenate results according to topological order of *H*.

Another standard optimization method that can be used here is hill-climbing. For each permutation of vertices *σ* we can compute the number of edges that are going back, i.e. *f*(*σ*).

Our goal is to minimize *f*(*σ*) over the permutation group. The simple version of the algorithm called HILLCLIMBINGSIMPLE (Algorithm 1) in every step swaps two randomly chosen vertices and checks if such a new permutation decreases the value of *f*. We can run this algorithm for each strongly connected component separately and concatenate results at the end.



The problem with HILLCLIMBINGSIMPLE is that even if algorithm stops there still could exist two vertices such that their swap decrease the target function. One possible way to find them is to iterate over all pairs of vertices and check if swapping any of them could improve the function *f*. However the time complexity of such method is *O*(|*V*|^2^|*E*|) because each call of COUNTBACKEDGES is proportional to the size of graph. The following Proposition allows to reduce this complexity.

#### **Proposition 2**

Let *σ*be some permutation of vertices of graph *G*=(*V*,*E*). We denote the permutation that came from *σ*by swapping *v* and *w* by *σ*(*v*⇔*w*). Let us assume, that we know the value of *f*(*G*,*σ*), furthermore *σ*(*v*)<*σ*(*w*) and *V* ^′^={*x*∈*V*:*σ*(*v*)≤*σ*(*x*)<*σ*(*w*). Then,

$$\begin{aligned} f\left(G, \sigma\left(v \leftrightarrow w\right)\right) &= f(G,\sigma) - \left|\{(w, x) \in E\ \mid\ x \in V' \}\right|\\ &\quad+ \left|\{(x, w) \in E\ \mid\ x \in V' \}\right| \\ &\quad+ \left|\{(v, x) \in E\ \mid x \in V' \}\right|\\ &\quad- \left|\{(x, v) \in E\ \mid\ x \in V' \}\right| \end{aligned} $$

Using Proposition 2 we can compute value of COUNTBACKEDGES for a given vertex in time proportional to number of edges *O*(|*E*|). Therefore, the time complexity of finding an optimal pair of vertices is *O*(|*V*||*E*|).

#### **Proposition 3**

Let *σ*be some permutation of vertices of a graph *G*(*V*,*E*), *v*_1_,*v*_2_,*w*_1_,*w*_2_∈*V* be pairwise distinct. If at least one of the following conditions is satisfied:

$$\begin{array}{*{20}l} \ & [\sigma(v_{1}), \sigma(v_{2})] \cap [\sigma(w_{1}), \sigma(w_{2})] = \emptyset \\ \text{or\ \ \ \ } &[\sigma(v_{1}), \sigma(v_{2})] \subset [\sigma(w_{1}), \sigma(w_{2})] \\ \text{or\ \ \ \ } & [\sigma(w_{1}), \sigma(w_{2})] \subset [\sigma(v_{1}), \sigma(v_{2})] \end{array} $$

where [*σ*(*v*),*σ*(*w*)] denotes the following set: {*σ*(*x*):*σ*(*v*)≤*σ*(*x*)≤*σ*(*w*)} then

$$\begin{aligned} f\left(G, \sigma(v_{1} \leftrightarrow v_{2})(w_{1} \leftrightarrow w_{2})\right) &= f\left(G, \sigma(v_{1} \leftrightarrow v_{2})\right)\\ &\quad+ f\left(G, \sigma(w_{1} \leftrightarrow w_{2})\right)\\ &\quad- f(G, \sigma) \end{aligned} $$

Using Propositions 2 and 3 we improve HILLCLIMBINGSIMPLE, so that it proceeds until there are no two vertices such that their swapping could decrease our target function (number of back edges).

Namely, for given *σ* we can compute the following set of triples:

$$I_{\sigma} = \{(v, w, c) \mid v, w \in V\ \wedge\ c = f(G, \sigma) - f\left(G, \sigma(v \leftrightarrow w)\right)\} $$

Hence, for each pair of vertices we know how much they can improve *f*(*σ*). Using Proposition 2, the complexity of computing *I*_*σ*_ is *O*(|*V*||*E*|). From Proposition 3 we know that if we choose (*v*_1_,*v*_2_,*c*_1_),(*w*_1_,*w*_2_,*c*_2_)∈*I*_*σ*_ such that *v*_1_,*v*_2_,*w*_1_ and *w*_2_ satisfy assumptions of Proposition 3 then,

$$f\left(G, \sigma(v_{1} \leftrightarrow v_{2})(w_{1} \leftrightarrow w_{2})\right) = f\left(G,\sigma\right) - c_{1} - c_{2}$$

Therefore we reduce our task to finding such a subset for {(*v*_*i*_,*w*_*i*_,*c*_*i*_)}⊆*I*_*σ*_, so that sum of *c*_*i*_ is maximal and (*v*_*i*_,*w*_*i*_),(*v*_*j*_,*w*_*j*_) satisfy assumptions of Proposition 3 for each *i*,*j*.

This problem could be solved by dynamic programming. Assume that all *v*_*i*_ are less than *n* where |*V*|=*n*, then by *d*[*i*][*j*] we denote the sum of *c*_*i*_ for optimal solution that includes only vertices that have number between *i* and *j* in order defined by permutation *σ*, namely *i*≤*σ*(*v*)≤*j*. In other words, *d*[*i*][*j*] is maximum value we can decrease function *f* permuting only vertices from *i*-th to *j*-th inclusive, that satisfy Proposition 3. Array *d* could be computed with use of the following Proposition:

#### **Proposition 4**

For a given *I*_*σ*_ defined above:

$${\begin{aligned} &d[i][j]\\ &= \left\{\!\! \begin{array}{cc} 0&\text{if~} i\leq j\\ \max\left(\max\limits_{i < k < j}\left(d[i][k] + d[k+1][j]\right), d[i+1][j-1] + V_{ij}\right)&\text{otherwise}\\ \end{array} \right. \end{aligned}} $$

Where *V*_*ij*_ is such that (*i*,*j*,*V*_*ij*_)∈*I*_*σ*_.

Having computed the array *d* we know the value that decreases *f*, but we also need to find a subset of *I*_*σ*_ that generates this value. This could be achieved by standard back-tracking scheme, i.e. for each *i*,*j* we store information whether *d*[*i*][*j*] comes from including some element of *I*_*σ*_ or it is concatenation of solutions for some *d*[*i*][*l*] and *d*[*l*+1][*j*]. Assume that the procedure called EXHAUSTIVESEARCH implements the process of finding *I*_*σ*_. Its time complexity is *O*(|*V*|^3^) and memory complexity is *O*(|*V*|^2^).



Finally, we can modify hill climbing approach of Algorithm 1 to use EXHAUSTIVESEARCH (Algorithm 2) every time when it fails to find two vertices that can decrease function *f*. This approach formalizes into Algorithm 3. The time complexity of Algorithm 3 is of the same order as Algorithm 2, i.e. *O*(|*V*|^3^).



### Functionality of TRANScendence tool

**Fig. 1:**
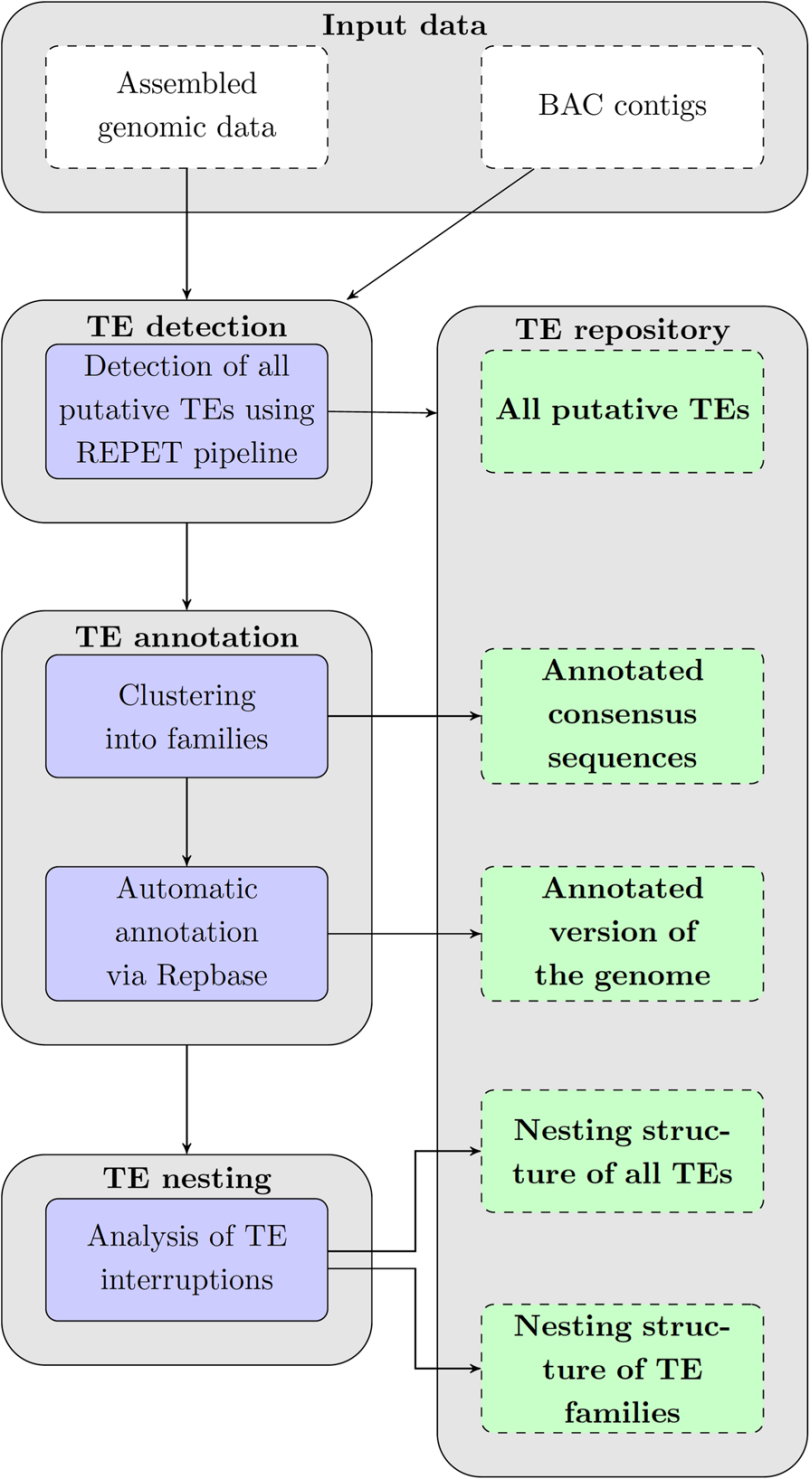
Overview of the TRANScendence tool

The interruption graph is constructed based on the nesting structure of all TEs detected in analyzed genome. TRANScendence tool aims in accurate de-novo detection and annotation of these TEs. The standard use-case consists of three steps: TE detection phase, TE annotation phase and TE nesting analysis, see Fig. 1. The workflow of our utility usually begins with a user uploading (through a web-based interface) a (possibly zipped) set of FASTA files, containing the genome of organism to be searched. The user then creates an ’experiment’ on the genome. Within the experiment, the genome gets automatically searched for TEs with the help of REPET pipeline [6].

The results of the detection phase are all putative transposable elements stored in the TE repository. Further in the annotation phase all elements found within the experiment, are automatically annotated (against Repbase), and are made available for the user to either view or download. Furthermore, the TEs get clustered into families, with consensus sequences for each family being computed, and annotated. The consensus sequences for detected TE families may be downloaded as well. For the convenience of users the additional output is an annotated version of the genome, in formats suitable for widely-used genomic browsers such as GBrowse [13] or Apollo [14].

In next step the tool analyzes the nesting structure of detected transposable elements. A graphical visualization of TE interruptions is generated, allowing the study of genomic sites with deeply nested TEs, as well as the display of more detailed informations about such sites. In addition to that, the tool presents the user with a graph of interruptions on TE family level, allowing for chronological study of TE families. Also an interruption matrix is generated (and presented for downloading by the user). To ensure the high quality of data stored in the repository the user is allowed to perform manual curation of all annotations. The useful option available to the user is to present his own uploaded genomes as ’public’, i.e. and viewable by other users of the system. Experiments performed by users may be shared with their consent as well. Using this mechanism, the database of *Medicago truncatula* TEs has been made available for public viewing.

Let us illustrate the functionality of the tool by exploring the TE landscape of the *Medicago truncatula* genome. *Medicago truncatula*, also called barrel medic is the primary model, or reference legume species for genomic and functional genomic research. The sequenced part of its genome (ca. 313 Mbp) was scanned for TEs and 121509 elements have been found and grouped into 2456 families. Approximately 80 Mbp have been found to be attributed to TEs

1356 families group 51740 class I TEs, while 59855 class II elements were classified into 803 families. Among DNA TEs 4907 elements are MITEs and the set of other TEs carrying terminal inverted repeats encompasses: 4475 Harbinger, 3181 hAT, 1015 Mariner/Tc1, 210 Enhancer/Suppressor mutator (En/Spm)-like TEs, 22657 MuDR elements and only 3 Polinton elements. It should be noted that in the latter case the homology to known Polintons from Repbase [7] is rather weak, so this annotation might require further curation. Finally 2557 elements were classified as Helitrons – this is, surprisingly, much more than in closely related species like *Lotus japonicus* [15].

Detected retrotransposons were divided into most abundant Gypsy superfamily (16440 TEs), Copia (15930 TEs), 26 ERV1-type repeats, 8195 L1 elements, and 134 RTE superfamily elements. TE detection phase is summarized with several statistics presented at Experiment’s results page.

**Fig. 2:**
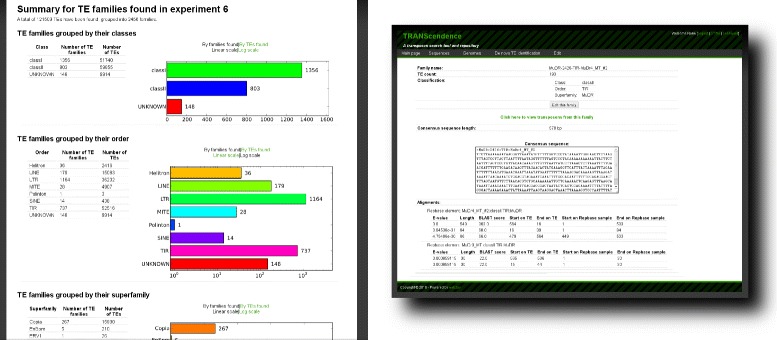
All found putative TEs are classified into classes, orders and superfamilies. Each TE family is annotated by BLASTing the consensus sequence against the Repbase content

In addition to the quantitative results which allow to compare genomes composition in various TE families, all the data stored in our database can be easily manipulated and visualized. The example of usage may be downloading the TE flanking sequences for designing the PCR starters. As stated above all putative TEs are automatically classified into orders and superfamilies. Moreover we annotate each detected TE family by aligning the family consensus sequence against elements stored in Repbase [7], see Fig. 2.

## Results and discussion

To validate the correctness of our method for TE chronology reconstruction we decided to perform tests on in silico generated data. This approach enables the quantification of accuracy of proposed algorithms. On the other hand the validation on real datasets is much more challenging, as there are very few available data concerning the evolutionary history of TE families.

### Validation for fixed model of evolution

We prepared a tool that generates interruption events, in form of tuples (*f* *a* *m* *i* *l* *y* *F* *r* *o* *m*,*f* *a* *m* *i* *l* *y* *T* *o*,*c* *o* *u* *n* *t*), in order to test our algorithms on artificial data and compare them to real data extracted from *TRANS*cendence.

We assume a model of disjoint, sequential periods of activity of *TE* families, i.e., for each family we randomly select the size of family *F*_*i*_ from the Poisson distribution and for each *TE* from family *i*, we create an edge to family *j* with probability equal to:

$$P(i, j) = \left\{ \begin{array}{cc} 0&\text{if}\ i \geq j\\ &\\ \frac{F_{j}}{|G|+\sum\limits_{k=1}^{i-1} F_{k}}&\text{if}\ i > j \end{array} \right. $$

Where |*G*| denotes the genome size.

To test the robustness of our method we add the noise modeled as random interruption events (e.g. 10% of noise corresponds to 10% of random edges in TE interruption graph).

The natural score to compare outcome of algorithms is the number of back edges. Since we generate the graph according to the model we know the order of vertices in advance. The following table summarizes the accuracy of our algorithms. The first three columns represent model parameters. Namely, number of TE families, mean number of TE in each family, percent of randomly generated edges (noise). Genome size is assumed to be 5·10^4^.

The HILLCLIMBING algorithm finds permutations with number of back edges that is very close to the number of back edges in the original order. On the other hand, quasi topological sort performs much worse.

We can also measure how much the order of vertices that we achieve differs from the original order. A natural choice for metric is the number of inversions (the sequence has an inversion where two of its elements are out of their original order). Let *σ* be a permutation of numbers from 0 to *N*, then

$$inv(\sigma) = |\{\left(\sigma(i), \sigma(j)\right)\ |\ \sigma(i) > \sigma(j)\ \text{and}\ i < j\}| $$

Table 1 summarizes the average number of inversions (number of inversions divided by number of families) for different model settings. Which could be interpreted as an average distance of a family from its true positon in the chronology. Note that for original order this measure is equal to zero, so the lower value the closer to the initial order we are. In the last column there is the metric for 10 HILLCLIMBING runs. The results demonstrate that HILLCLLIMBING quite well approximates the original order.

**Table 1:** Accuracy measured in number of inversions for different model settings

No. Families	Mean	Noise	Quasi topological	Average hillClimbing
200	50	25%	14.6	8.8
200	50	50%	18.8	12.5
200	100	50%	11.0	4.5
300	100	15%	10.3	3.7
300	100	25%	12.2	4.5
300	100	50%	13.6	5.8
500	200	15%	10.9	2.6

### The chronology of TEs mobilization in *Drosophila melanogaster* and human genomes

To examine TRANScendence functionality, and accuracy of proposed chronology reconstruction algorithms, we performed analyses on *Drosophila melanogaster* and human genome.

First, we used our tool for defragmenting nested TEs insertions and creating an interruption matrix. Columns and rows of the matrix represent chosen TEs subfamilies. Interruption events between them, i.e. mobile elements from one family nested into element of the other were counted and stored in the matrix.

In the case of *Drosophila melanogaster* raw interruption matrix as generated by TRANScendence tool has been preprocessed as follows. We focused our analysis on LTR elements having significant similarity to some consensus sequences from the REPbase, belonging to superfamilies: Gypsy, Copia, Jockey, BEL, P, RLX, CR1, Loa, R1 and Mariner/Tc1. After preprocessing we applied Algorithm 3 to recover the order of mobilizations periods.

Matrix with topologically sorted TEs families is lower triangular, which clearly demonstrate that we successfully reconstructed the evolutionary history. However, it should be noted that such perfect linear ordering of disjoint TEs activities had been obtained for smaller subfamilies of elements sharing the same consensus sequence in REPbase. More careful analysis of outcomes shows that representatives of some families, like *Q* *U* *A* *S* *I* *M* *O* *D* *O*_*I*_, are widely spread throughout the timeline. It indicates that these LTR’s families were mobilized during the same time period or were mobilized repeatedly. Especially the latter behavior of TE activity is widely recognised. Recently proposed model of TEs proliferation proved that the subtle interplay between environmental stress and the host genome provoke bursts of TEs mobilization [8].

The alternative method for chronology reconstruction is based on TE sequences divergence. We compared our results with the only one study performed for *Drosophila melanogaster* in [16]. The age distribution of considered TE families is summarized in Fig. 1 of [16]. Inside LTR superfamily there are three families (invader2, micropia and Tabor) classified as older, while the age of others are smaller and indistinguishable. Note, that the significance of this result is affected by small number of analysed TE copies from these three families. Our analysis suggests the interleaving periods of activities for all considered LTR families. In the case of much older non-LTR TEs our methods resulted in linear ordering of TE mobilisation periods different than ordering of TEs age. It may suggests the significant discrepancy between the age of the TE families and the periods of their mobilization. It may happen e.g. if relatively old TE families were active for a long interval or mobilized several times during their evolution. It should be noted that our approach, in contrast to methods of age dating based on sequence divergence, allows to compare TEs from different families and therefore can detect more subtle relationships.

**Fig. 3:**
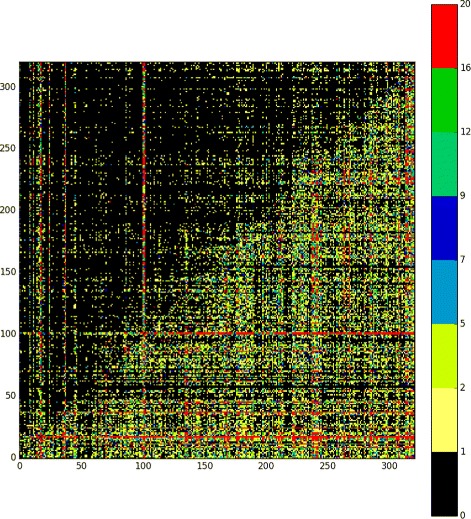
Ordered interruption matrix for 320 human TEs families

Focusing on human genome, we have performed a study, comparing the chronology derived by our tool with the chronology obtained from the only other tool which uses TE interruptions to recover TE chronology, that we are aware of [1], see Fig. 3 for ordered matrix of 320 human TEs families. Following [1] (Table 2) we have performed a reconstruction of the well-known L1PA family of transposons. The current consensus (based on phylogenetic study) is that the L1PA family is chronologically ordered by number (L1PA17 being oldest and L1PA2 youngest), with L1Hs, the currently active family being youngest of them all. The ordering we have recovered is in remarkable agreement with that derived from phylogeny, deriving from it by 1 inversion, compared to two inversions in [1]. The one inversion found by our algorithm (L1PA14/L1PA13) is also repeated in [1], which suggests the possibility that it might be an artifact of the phylogeny-based chronology.

**Table 2:** Accuracy (measured as number of back edges) of discussed algorithms for different model parameters

No. Families	Mean	Noise	No. Edges	Original order	quasi-topological	hillClimbing
200	50	25%	4608	763	943	743
200	50	50%	6137	1502	1763	1444
200	100	50%	18289	4514	5266	4521
300	100	15%	21026	2437	3242	2480
300	100	25%	24255	4063	5073	4102
300	100	50%	32323	8086	9184	8075
500	200	15%	98521	11324	13876	11534

## Conclusions

Predictably, the lack of easily usable TE annotation pipelines hinders the comparative genomics of TE families. Especially the problem of establishing chronologies of activity of various TE families on relation to one another remains elusive. The standard approach of reconstructing the phylogenetic trees of TEs is somewhat hindered by the dispensable status of TEs. Because they are not conserved, they are vulnerable to large-scale genomic deletions and other rearrangements. This causes TE copies to be mangled and fragmented, which presents a difficulty for standard phylogenetic tools [17, 18], as they have mostly been developed with (much better-conserved) genes in mind. As such, the chronologies obtained from sequence-similarity-based tools are not fully trusted, and could stand to be verified with other methods.

One such attempt at verification has already been performed for human genome [1] establishing chronology based on the insertions of TEs within each other. The approach however, while being a significant improvement over the previous methods, depends on a preexisting TEs database, and employs an ad-hoc TE defragmentation method. Obviously, the state-of-the-art utilities for de-novo TE detection and analysis could widen the scope of application of this method, as well as produce better results.

To this end we focused our tool on obtaining interruption matrices of TEs (that is, data representing how TEs nest in each other), and provides an option of visualizing the interruptions graph – which constitute the useful tool for assessing the periods of activity of TE families, as well as for their dating.

Moreover, we present original approach to infer the evolutionary history of TEs families. Our algorithms find the chronology in time *O*(|*V*|^3^) and we justified their correctness on in silico datasets.

The validation on real dataset has been performed on TE families from *Drosophila melanogaster* and human genome.

A possible direction of further research is taking into account overlapping periods of activity. This would relax the constraint on the matrix to be lower triangular and significantly extend the existing approaches. Moreover, the bayesian Markov chain Monte Carlo inference method could be applied to appropriately define the evolutionary model. In such approach we can define a stochastic process with configuration space corresponding to periods of activity of specific TE family.
